# Pituitary stalk changes on magnetic resonance imaging following pituitary adenoma resection using a transsphenoidal approach

**DOI:** 10.3389/fneur.2023.1049577

**Published:** 2023-01-27

**Authors:** Huijian Zhang, Shuai Zhang, Mingchao Shang, Jiaxing Wang, Liangfeng Wei, Shousen Wang

**Affiliations:** ^1^Department of Neurosurgery, 900th Hospital of Joint Logistic Support Force, Fuzhou, Fujian, China; ^2^Department of Neurosurgery, Huanggang Central Hospital, Huanggang, Hubei, China; ^3^Fuzong Clinical Medical College of Fujian Medical University, Fuzhou, Fujian, China

**Keywords:** diabetes insipidus, microsurgery, neurohypophysis, pituitary adenoma (PA), pituitary stalk, transsphenoidal surgery

## Abstract

**Objective:**

We aimed to investigate the magnetic resonance imaging (MRI) findings and clinical significance of position and changes in morphology of the pituitary stalk following pituitary adenoma (PA) resection using a transsphenoidal approach.

**Methods:**

We collected clinical and MRI data of 108 patients with PA after transsphenoidal surgery. Diameter, length, and coronal deviation of the pituitary stalk were measured pre-, post-, and mid-term post-operatively, to observe pituitary stalk morphology.

**Results:**

Of 108 patients, 53 pituitary stalks were recognisable pre-operatively. The angle between the pituitary stalk and the median line was 7.22°-50.20° (average, 25.85°) in 22 patients with left-sided pituitary stalks and 5.32°-64.05° (average, 21.63°) in 20 patients with right-sided pituitary stalks. Of 42 patients with preoperative pituitary stalk deviation, 41 had an early postoperative recovery and 1 had increased deviation. In the mid-term postoperative period, 21 of 42 patients had pituitary stalks located centrally. In 53 patients, the pituitary stalk length was 1.41–11.74 mm (mean, 6.12 mm) pre-operatively, 3.61–11.63 mm (mean, 6.93 mm) in the early postoperative period, and 5.37–17.57 mm (mean, 8.83 mm) in the mid-term postoperative period. In the early postoperative period, 58 (53.70%) patients had posterior pituitary bright spots (PPBS) and 28 (25.92%) had diabetes insipidus (DI).

**Conclusion:**

Pre-operatively, the pituitary stalk was compressed and thinned. Post-operatively, it could be stretched to a “normal state”, and its position showed a gradual centring trend. Post-operatively, the length of the pituitary stalk gradually increased. The PPBS in the early postoperative period negatively correlated with postoperative DI.

## 1. Introduction

The pituitary stalk runs obliquely in the centre of suprasellar cistern, connecting the hypothalamus and pituitary glands, and plays an important role in maintaining the normal physiological function of the pituitary gland. In patients with pituitary adenoma (PA), the pituitary stalk moves up, deflects, deforms due to the influence of the tumour, and is squeezed at the sella phrenic foramen, thereby affecting the pituitary portal system and thus changing the pituitary function (1). In microsurgical transsphenoidal surgery (TSS), the pituitary stalk may also be damaged during surgery or tumour resection, which may lead to early postoperative

diabetes insipidus (DI) (2, 3). DI is a common complication after microsurgical TSS (incidence rate, 1–67%) (4). Following microsurgical TSS, the position of the lower end of the pituitary stalk changes accordingly. At 3–4 months post-operatively, gelfoam and effusion that fill the tumour cavity during surgery are gradually absorbed (5). When the sellar diaphragm further descends, the pituitary stalk can be pulled due to the descent of the sellar diaphragm and pituitary gland, resulting in a change in shape to the pituitary stalk, and even a functional change. This change in morphology concerning the pituitary stalk has rarely been discussed. We aimed to observe changes to the pituitary stalk in patients who had undergone microsurgical TSS and to analyse this change using pre- and post-operative magnetic resonance imaging (MRI).

## 2. Materials and methods

### 2.1. General information

In this retrospective study, we analysed 108 patients (45 males, 63 females; age range, 18–67 years; average age, 46 years) from the Department of Neurosurgery at the 900^th^ Hospital who had undergone PA surgery from January 2015 to December 2019 *via* a microsurgical TSS approach. Among these patients, 11 had pituitary micro-adenomas (maximum diameter, <1 cm), 76 had pituitary macro-adenomas (maximum diameter, 1–4 cm), and 21 had giant PAs (maximum diameter, >4 cm). Inclusion criteria comprised patients: (i) who were pathologically diagnosed with PAs and who had undergone microsurgical TSS by the same physician; (ii) with complete preoperative MRI data; and (iii) who had MRI re-examination time points during the early postoperative period (within 1 week), the mid-term postoperative period (4–8 months), and the late postoperative period (8–12 months) as well as data from at least two postoperative MRI. Exclusion criteria comprised patients with: (i) a history of preoperative radiotherapy and chemotherapy, (ii) a history of reoperation, or (iii) an unclear image. This study was approved by the Hospital Ethics Association, and all 108 patients had provided written informed consent.

### 2.2. MRI

A Siemens 3.0T magnetic resonance machine (Siemens AG, Germany) was used to perform the axial and sagittal T1WI, axial and coronal T2WI, and three-dimensional contrast-enhanced sequences of pituitary MRI. Conventional MR scanning parameters were as follows: T1WI, involving a fast spin echo sequence; TR, 400–500 ms; TE, 8–15 ms; and three excitations. T2WI was performed with a TSE sequence, TR 3,000 ms, TE 83–98 ms, and two excitations. The scanning field of view (FOV) was as follows: 180 × 180 mm, matrix: 320–384 × 240–252, axial scanning layer thickness 1.0 mm, and layer spacing 6.5 mm. In the coronal and sagittal scans, the slice thickness was 1.0 mm and slice distance was 2.50 mm. Following a plain scan, a three-dimensional enhanced scan was performed with the body position unchanged. A gadolinium pentamine injection (dose, 0.2 mmol/kg body weight) was used as the contrast agent. The scanning parameters were the same as those listed above. Each patient had at least one preoperative scan and two postoperative scans.

### 2.3. Surgical method

Patients were laid in the supine position with the head tilted back approximately 20°. A right nostril approach was used. Under the operating microscope, the nasal septum mucosa was cut transversely 2.5 cm from the tip of the nose and separated to the anterior wall of the sphenoid sinus. At the junction of the vertical plate of the ethmoid bone and the anterior wall of the sphenoid sinus, the bone of the nasal septum was broken and pushed away to the opposite side. A nasal dilator was used to stretch the nasal septum mucosa on both sides and expand the visual field of the anterior wall of the sphenoid sinus. After having confirmed the ostium of the sphenoid sinus, the anterior wall of the sphenoid sinus was ground off, the sphenoid sinus was entered, the septum of the sphenoid sinus was ground off, the sellar base was identified and exposed, and a bone window was opened with a diameter of 1.5–2 cm in the anterior wall of the sellar base. An X-cut of the dura mater at the bottom of the saddle was made to observe the tumour tissue. The tumour was carefully removed using a suction device, a scraping ring, and tumour forceps in a back, top, and front order. The surrounding normal pituitary tissue was fully identified and preserved. A gelatin sponge was used to stem the bleeding. Finally, freeze-dried fibrin glue (Shanghai Laishi 2 mL/branch) was used to fix the tissue. If cerebrospinal fluid leakage occurred intraoperatively, the sellar base was closed with an artificial dura. Osseous reconstruction of the sellar floor and foreign body packing in the sphenoid sinus cavity were not performed. Both nasal cavities were filled with surgical polyvinyl alcohol sponge and removed on post-operative day three.

### 2.4. MRI evaluation and diagnosis of postoperative DI

At least two neurosurgeons and one radiologist, including the operator, evaluated the MRI data. The diameter of the horizontal pituitary stalk at the upper edge of the pituitary gland was measured on coronal T1WI enhanced images using the Picture Archiving and Communication System (PACS). The distance from the optic chiasm plane to the upper edge of the pituitary gland was measured on the T1WI sagittal plane as the length of the pituitary stalk. The deflection angle of the pituitary stalk was measured on the coronal image of the pituitary stalk, and posterior pituitary bright spots (PPBS) were observed. Each patient's urinary volume was recorded every 60 min and every 24 h post-operatively. If the urine volume exceeded 300 mL/h for three consecutive hours or exceeded 3,500 mL for 24 h, we considered the patient to have postoperative DI, and desmopressin acetate was administered as treatment and electrolyte changes were monitored. Postoperative DI was diagnosed when the urine volume was >300 mL/h for 3 h consecutively or >3,500 mL for 24 h and when desmopressin acetate was effective (6–8).

### 2.5. Statistical analysis

SPSS 22.0 software (SPSS Inc., Chicago, Illinois, USA) was used for statistical analysis. Measurement data were expressed as mean ± standard deviation. Data conforming to normal distribution were analysed using paired *t-*tests. Counted data are expressed as a percentage, using a χ^2^ test (or Fisher's exact test), with statistical significance set at *P* < 0.05.

## 3. Results

Among 108 patients who had undergone trans-nasal PA surgery, there were 45 males and 63 females ranging in age from 18 to 67 years (average age, 46 years). Of these, there were 11 pituitary micro-adenomas (maximum diameter, <1 cm), 76 pituitary macro-adenomas (maximum diameter, 1–4 cm), and 21 giant PAs (maximum diameter, >4 cm). Early postoperative DI occurred in 28 (25.92%) patients, all of whom recovered after symptomatic treatment.

### 3.1. Changes in pituitary stalk position

**Figure 1:**
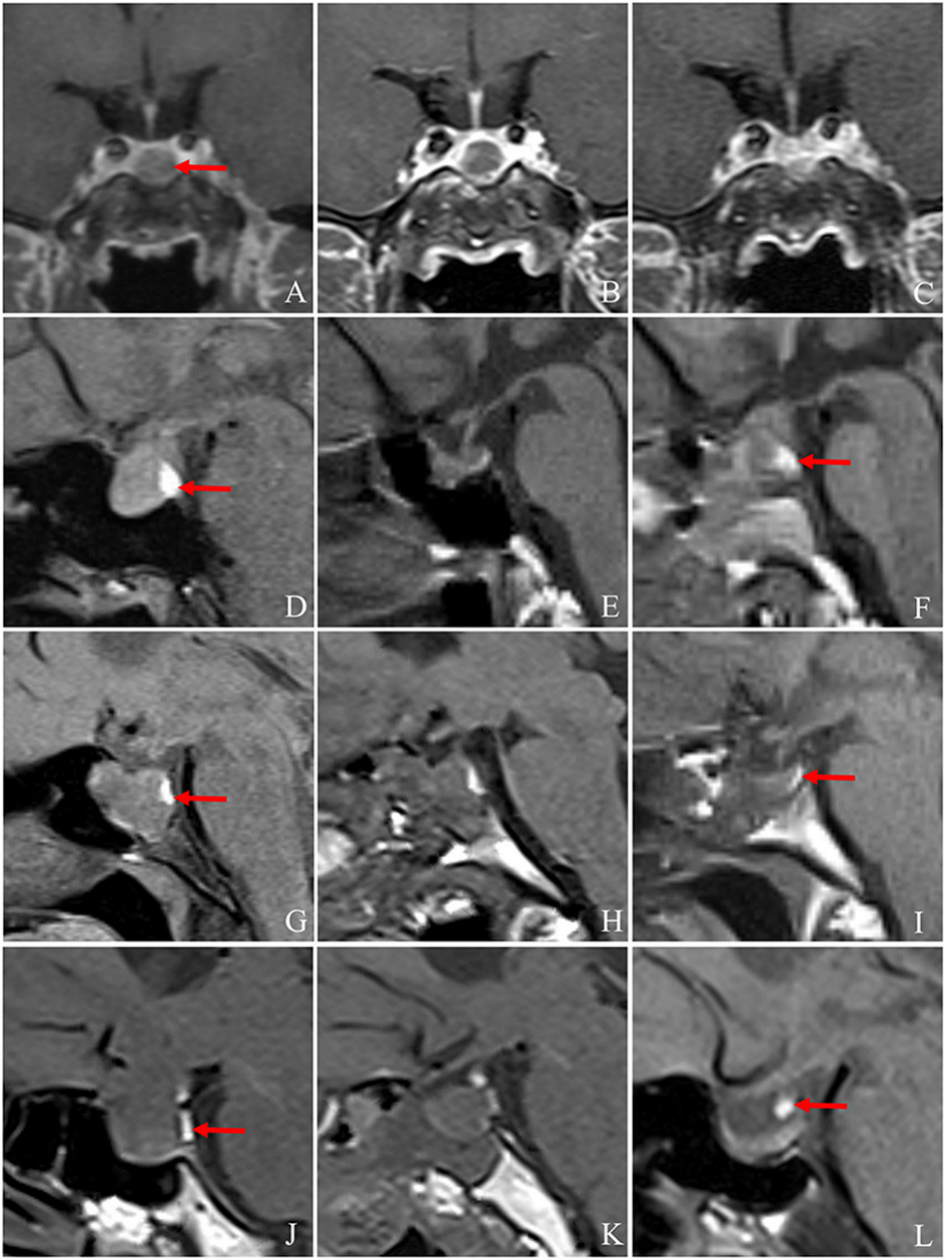
Changes in the pituitary stalk and PPBS pre- and post-operatively. **(A)** The pituitary adenoma is located directly below the pituitary stalk (arrow) pre-operatively, and the pituitary stalk is centred without deviation. **(B, C)** MRI showed that the pituitary stalk was in the middle on the 6th day and the 4th month post-operation. **(A–C)** The pituitary stalk is centred on MRI pre- and post-operatively. **(D–F)** The PPBS of a 42-year-old female patient persisted pre-operatively and on the third day and at 4 months post-operatively, and the signal intensity decreased slightly post-operatively. The position of the posterior pituitary lobe decreased to the sellar base at 4 months post-operatively, and no obvious abnormality was found in the pituitary stalk (arrow indicates PPBS). **(G–I)** A 44-year-old female patient with persistent PPBS pre-operatively and on the 1st day and at 4 months post-operatively. The volume of PPBS decreased significantly. The position of the posterior pituitary lobe decreased to the sellar base 4 months post-operatively. No obvious abnormality was found in the pituitary stalk. The patient had no diabetes insipidus post-operatively (arrow indicates PPBS). **(J–L)** T1WI of a 59-year-old female patient pre-operatively, on the 2nd day post-operatively, and at 4 months post-operatively, respectively. Pre-operatively, two sections of PPBS are observed as being located behind the tumour. Post-operatively, PPBS is located in the lower part of the pituitary stalk, and its volume is larger than that pre-operatively. The patient did not have diabetes insipidus post-operatively (arrow indicates PPBS). MRI, magnetic resonance imaging; PPBS, posterior pituitary bright spots.

The pituitary stalk was recognisable in 53 patients. Pre-operatively, the pituitary stalk was approximately centred in 11 patients, who either had micro-adenomas or small adenomas (diameter, approximately 1 mm), or the tumour was biassed to one side but was small and did not lead to displacement of the pituitary stalk, or the tumour was located directly below the pituitary stalk and did not move the pituitary stalk (Figures 1A–C). The position of the pituitary stalk did not change significantly post-operatively. Pre-operatively, the pituitary stalk deviated to the left in 22 patients, and the angle between the pituitary stalk and the median line was 7.22°-50.20° (average, 25.85°), whereas the angle between the pituitary stalk and the median line was 5.32°-64.05° (average, 21.63°) in 20 patients. There were 42 patients with pituitary stalk deviation pre-operatively, of whom 41 had an early postoperative recovery, with 9 patients having left-sided deviations. The angle between the pituitary stalk and the median line was 14.10°-31.34° (average, 22.33°) pre-operatively. The angle between the pituitary stalk and the median line was 6.66°-36.10° (average, 16.58°) at the mid-term postoperative period. One patient had an increased deviation. In the mid-term postoperative period, six patients still had a left-sided deviation, and the angle between the pituitary stalk and the median line was 10.89°-22.12° (average, 17.53°). The angle between pituitary stalk and the median line ranged from 4.34° to 25.70° (mean, 12.11°). The coronal deviation of the pituitary stalk was changed in 53 patients pre-operatively, early post-operatively, and at the mid-term postoperative period. We observed that the pituitary stalk gradually recovered in the mid-term postoperative period.

### 3.2. Morphological changes in the pituitary stalk

Among 108 patients, 53 pituitary stalks could be clearly identified pre-operatively, and some pituitary stalks were squeezed into an “S” shape or bent into a “C” shape. The diameter of the upper edge of the pituitary stalk was 1.08–3.89 mm (mean, 2.38 mm) in 53 patients pre-operatively, and there was no obvious thickening of the pituitary stalk. The diameter of the upper edge of the pituitary stalk was 1.29–3.43 mm (mean, 2.30 mm) in the mid-term post-operative period. The diameter of the pituitary stalk post-operatively showed a tendency to return to a normal shape compared with its preoperative shape. Pre-operatively, the pituitary stalk was relatively thickened or compressed up to the mid-term post-operative period and correspondingly stretched or thickened at the baseline up to 2 mm.

In the sagittal position, the distance from the visual cross plane of the pituitary stalk to the upper edge of the pituitary gland comprised the length of the pituitary stalk. In some patients, the pituitary stalk was too oblique to be measured. The length of the pituitary stalk in 42 patients was 1.41–11.74 mm (average, 5.57 mm) pre-operatively, 3.61–11.63 mm (average, 6.93 mm) in 48 patients in the early postoperative period, and 5.37–17.57 mm (average, 8.76 mm) in 48 patients in the mid-term postoperative period. The diameter and length of the pituitary stalk were measured in 42 patients with PA pre- and post-operatively. The results show that there were no statistically significant differences in the diameter of the pituitary stalk pre-operatively and in the mid-term postoperative period (*P* = 0.357). The length of the pituitary stalk in the mid-term postoperative period was statistically significantly longer than in the preoperative period (*P* < 0.01) (Table 1). Among 53 patients, the pituitary stalk could not be identified in 23 patients in the early postoperative period, and eight patients in the mid-term postoperative period. The length of the pituitary stalk was 2.73–11.64 mm (mean, 6.69 mm) in 30 patients in the early postoperative period and 5.13–12.79 mm (mean, 9.14 mm) in 45 patients in the mid-term postoperative period. The length of the pituitary stalk gradually stretched from the early postoperative period to the mid-term postoperative period. The “C”- and “S”-shaped pituitary stalks gradually returned to normal after PA resection. The diameter of the pituitary stalk did not change significantly and its length gradually extended post-operatively.

**Table 1:** Comparison of pituitary stalk diameter and length in 42 patients with pituitary adenoma before and after operation.

**Group**	**Pituitary stalk diameter (mm)**	**Pituitary stalk length (mm)**
Preoperative	2.37 ± 0.83	5.57 ± 2.21
Mid-term postoperative period	2.22 ± 0.50	8.76 ± 2.68
***T***	0.928	2.500
***P***	0.357	0.000

### 3.3. The posterior pituitary bright spot

Among the patients with PAs, 58 (53.70%) patients showed PPBS pre-operatively, five patients' PAs disappeared in the early postoperative period, and 53 patients showed bright spots in the mid-term postoperative period. PPBS can persist pre-operatively, in the early postoperative period, and in the mid-term postoperative period (Figures 1D–F). A small number of PPBS disappeared in the early postoperative period (Figures 1G–I), and some PPBS moved to the lower part of the pituitary stalk (Figures 1J–L). There were 46 (49.07%) patients with PPBS lobe in the early postoperative period, 41 (25.92%) patients had DI in the early postoperative period, most of which were transient DI within 1 week post-operatively, and the urine volume was approximately 3,500 mL per day. The incidence of DI in patients with negative PPBS in the early postoperative period was 48.3%, which was slightly higher than that in patients with positive PPBS (28.3%), and the difference was statistically significant (*P* = 0.038), as shown in Table 2. In patients with PA, PPBS can be ectopic to the lower part of the pituitary stalk under the influence of the tumour and can increase or disappear post-operatively. PPBS presenting in the early postoperative period were found to be related to postoperative DI.

**Table 2:** Relationship between early postoperative diabetes insipidus and PPBS.

**Group**	**DI**	**Non-DI**	**χ^2^**	***P***
PPBS(+)	13	33	4.303	0.038
PPBS(−)	28	30		

DI, diabetes insipidus; PPBS, posterior pituitary bright spots.

## 4. Discussion

The pituitary stalk runs obliquely in the centre of the suprasellar cistern. Most normal pituitary stalks are located centrally, but the pituitary stalk can show physiological deviation (9). However, PA does not necessarily lead to pituitary stalk deviation. Therefore, when pituitary stalk deviation is observed clinically, it can only be used as an indirect sign of PA, even when the pituitary stalk is located centrally. PA cannot be excluded as the pituitary gland is large and the pituitary stalk can be compressed; therefore, MRI often cannot distinguish the pituitary stalk.

The pituitary stalk tends to recover to a central position after PA surgery, and gradually shifts with the decline of the sellar diaphragm from the early postoperative period to the mid-term postoperative period. In this study, 21 of 42 (50%) patients had pituitary stalk deviation that was largely located centrally in the mid-term postoperative period, and other pituitary stalk deviations also resolved. Part of the pituitary gland may be biassed to one side post-operatively due to tumour compression, and the position of the pituitary stalk can be difficult to restore to a central position.

The average diameter of the lower end of the pituitary stalk measured using MRI was 1.9–2.3 mm, and the average length of the pituitary stalk was 9.6 mm (10–13). The average diameter of the pituitary stalk was 2.37 mm pre-operatively and 2.22 mm in the mid-term postoperative period. The average length of the pituitary stalk was 5.57 mm pre-operatively and 8.76 mm in the mid-term postoperative period. From the preoperative to the mid-term postoperative period, the length of the pituitary stalk showed a “stretched” state; however, the pituitary stalk diameter did not become thinner due to the extension of the pituitary stalk, but gradually returned to a normal diameter. This indicated that following PA surgery, in addition to the morphological remodelling of the pituitary gland due to the release of compression, the pituitary stalk also had similar changes. The posterior pituitary usually shows a high signal on T1WI, which generally indicates PPBS. Currently, it is considered to involve a short T1 signal of arginine vasopressin stored in the posterior pituitary (14). The presence of PPBS can indirectly indicate that the neurohypophysis functions well, and the disappearance of PPBS is helpful in diagnosing DI. If the location of PPBS in patients with PAs can be accurately determined pre-operatively, it is likely to be beneficial in protecting PPBS during surgery, so as to reduce the damage of neurohypophysis. For patients with negative PPBS, it is difficult to identify the neurohypophysis, which then carries a greater surgical risk and a greater possibility of postoperative DI.

Patients with negative PPBS pre-operatively were more likely to have postoperative DI (10, 15). A high signal in the posterior pituitary reflects storage of arginine vasopressin, but some patients with negative PPBS did not have DI. It appeared that synthetic arginine vasopressin and its consumption reached a dynamic balance. We found no significant correlation between PPBS and DI in the early postoperative period. Considering that the time of MRI re-examination in the early postoperative period was not entirely consistent, negative PPBS in the early postoperative period may not necessarily reflect a lack of arginine vasopressin in a patient. A patient may have negative PPBS due to the release of stored arginine vasopressin into the blood, or the patient's synthetic arginine vasopressin and consumption reaching a dynamic balance, without showing DI. Pre-operatively, the pituitary stalk was relatively thickened or compressed under the influence of PA. It can be curled up into a “C” or “S” shape and when part of it is close to the sellar diaphragm, it is not readily recognisable. In the mid-term postoperative period, depression of the pituitary stalk was relieved, the diameter of pituitary stalk returned to a relatively normal state, and the pituitary stalk was curled up and gradually extended. PA can lead to deviation of the pituitary stalk. After PA resection, the pituitary stalk tended to reset centrally, and the length of pituitary stalk gradually stretched with the decrease in the sellar diaphragm until an empty sellar emerged. An early PPBS presentation after PA resection is related to early postoperative DI (16).

## 5. Conclusion

Based on observation of our single-centre patient cohort, we found that the pituitary stalk of patients prior to TSS was compressed and thinned, and it could be stretched or thickened to a normal state post-operatively. Post-operatively, the position of the pituitary stalk gradually changed from being slanted to being centred. Compared with the preoperative length, the length of the pituitary stalk gradually increased from 1 week to 3 months post-operatively. In addition, MRI within 1 week post-operatively showed a correlation between PPBS and postoperative DI and that patients with negative PPBS were more likely to have DI.

## Data availability statement

The original contributions presented in the study are included in the article/supplementary material, further inquiries can be directed to the corresponding author.

## Ethics statement

The studies involving human participants were reviewed and approved by Ethics Association of 900th Hospital of Joint Logistic Support Force. The patients/participants provided their written informed consent to participate in this study. Written informed consent was obtained from the individual(s) for the publication of any potentially identifiable images or data included in this article.

## Author contributions

HZ, SZ, MS, and SW conceived and designed the study and wrote the manuscript. LW and JW analysed the data and prepared the tables. All authors contributed to the article and approved the submitted version.
